# New prediction models for gross energy of pig urine using urinary nitrogen concentration and body weight: technical note

**DOI:** 10.5713/ab.25.0097

**Published:** 2025-05-12

**Authors:** Hyunseok Do, Bokyung Hong, Jeonghyeon Son, Noa Park, Beob Gyun Kim

**Affiliations:** 1 Department of Animal Science, Konkuk University, Seoul, Korea; 2 Department of Animal Science, North Carolina State University, Raleigh, NC, USA

**Keywords:** Body Weight, Gross Energy, Models, Nitrogen, Swine, Urine

## Abstract

The objectives were to evaluate previous equations for estimating gross energy (GE) of pig urine using urinary nitrogen (N) and to develop novel equations for estimating GE concentrations of pig urine. A total of 136 urine samples were obtained from pigs fed 18 diets in 2 experiments. The samples were analyzed for GE and N concentrations. The accuracy of previous equations was assessed by regressing the measured values minus the predicted values for urinary GE concentration on the predicted values centered to the mean. Novel equations for estimating the urinary GE concentration were developed using urinary N concentration and body weight (BW) as independent variables. The previous equations overestimated (mean bias; p<0.001) urinary GE concentrations and the overestimation was more pronounced for the low-GE urine samples (linear bias; p<0.001). The novel equations developed in the present work were: urinary GE concentration (kcal/kg) = −7.51+12.83×urinary N (r^2^ = 0.92 and p<0.001) and −16.33+14.00×urinary N+0.192×BW−0.030×urinary N×BW (R^2^ = 0.92 and p<0.001) where N as g/kg and BW as kg. Overall, the previous equations overestimate urinary GE, particularly for low-GE urine of pigs. Gross energy concentrations in urine can be fairly accurately estimated using urinary N concentration and BW.

## INTRODUCTION

Dietary energy contents represent the greatest proportion of feed cost in the swine industry. Therefore, precise determination of energy values of diets and feed ingredients is critical for pig diet formulations [1]. In the procedures of determining metabolizable energy (ME) or net energy (NE) of feeds, pig urine is quantitatively collected and the gross energy (GE) concentration of urine is determined [2]. The GE concentration of urine is determined after drying urine using a freeze dryer [3] or a drying oven [4]. However, direct determination of GE in liquid urine is not feasible whereas urinary nitrogen (N) concentrations can be relatively easily determined.

Two equations for estimating urinary GE have been suggested based on the urinary N of pigs [5,6]. However, the accuracy of the previous equations has not been validated. Therefore, the objectives of the present study were to evaluate the previously published equations using data from pig urine with varying N concentrations and to develop novel equations for estimating urinary GE concentration using urinary N concentration and body weight (BW) as independent variables.

## MATERIALS AND METHODS

### Animals, diets, and housing

A total of 136 urine samples were employed to evaluate the previous prediction equations (Table 1) published in the literature [5,6] and to develop novel equations. The urine samples were obtained from pigs fed 18 diets in 2 experiments [7,8]. All experiments were conducted using crossbred barrows (Landrace×Yorkshire) under the same experimental conditions. At the beginning of each period, BW of pigs was measured to determine feed allowance. The BW of pigs in Exp. 1 and 2 ranged from 6.0 to 17.7 kg and 28.8 to 111.4 kg, respectively (Table 2). The concentrations of crude protein (CP) in the experimental diets ranged from 8.0% to 22.7% (as-is basis). In both experiments, pigs were individually housed in metabolism crates equipped with a feeder, a fully slatted plastic floor, and a urine tray, allowing for the total and separate collection of urine and feces from each pig.

### Feeding and sample collection

In Exp. 1, daily feed allowance was calculated as 5.0% of initial BW of nursery pigs and three equal meals were provided at 08:00, 12:30, and 17:00 h. In Exp. 2, daily feed allowance was calculated as 3.0 times the ME requirements for maintenance (i.e., 197 kcal of ME per kg of BW^0.60^; [9]) based on the initial BW of the pigs in each period and the ME of the experimental diets. The daily feed allowance was divided into two equal meals and provided at 08:00 and 17:00 h. Each period of Exp. 1 consisted of a 4-day adaptation period and a 4-day collection period. Each period of Exp. 2 consisted of a 5-day adaptation period and a 5-day collection period. In Exp. 1, urine samples were collected in a bucket containing 6 *N* HCl for N preservation from day 5 at 14:00 h to day 9 at 14:00 h in which the amounts of 6 *N* HCl were determined based on the literature [10]. In Exp. 2, urine samples were collected in a bucket containing 6 *N* HCl from day 6 at 14:00 h to day 11 at 14:00 h. At the end of the urine collection, a urine sample was filtered by a cotton sheet and collected in a 200-mL bottle and immediately stored at −20°C for analyses.

### Chemical analyses

The 136 urine samples were analyzed for N using method 990.03 of the AOAC [11]. Thawed urine samples were added to the cotton ball and lyophilized to analyze GE using a bomb calorimetry (Parr 6400; Parr Instruments, Moline, IL, USA). The analysis of urinary GE was adapted from Kim et al [12] with minor modifications. Approximately 3 mL of urine was added to a cotton ball (0.2 g) placed in an iron container weighing approximately 14 g. The iron container with the cotton ball and the urine was lyophilized and the weight of the lyophilized samples was recorded again. The weight of the iron container was subtracted from the weight of lyophilized iron container with the cotton ball and the urine. Subsequently, the weight of cotton ball and the lyophilized urine was used to analyze GE concentration. The GE concentration of blank cotton ball was also determined to enable the calculation of GE in urine. The GE of the blank cotton ball was subtracted from the total GE of the cotton ball containing urine to calculate the GE of urine.

### Statistical analyses

The accuracy of the previous equations [5,6] for urinary GE were tested by regression analysis using the REG procedure of SAS (SAS Institute, Cary, NC, USA). In the regression model, the measured minus predicted urinary GE was the dependent variable and the predicted urinary GE minus the mean predicted urinary GE was the independent variable. In the linear regression, the intercept and the slope represented a mean bias and a linear bias, respectively. The CORR procedure of SAS was performed to determine correlation coefficients among urinary GE concentration, urinary N concentration, urinary GE-to-N ratio (GE:N), BW, and dietary CP. Novel equations for estimating the urinary GE concentration was developed by the REG procedure of SAS using urinary GE concentration as a dependent variable and the urinary N concentration and BW as independent variables. Statistical significance was declared when a p-value was less than 0.05.

## RESULTS

The urinary N and GE concentrations ranged from 1.06 to 15.55 g/kg and 2 to 187 kcal/kg, respectively (Table 2). The urinary GE concentration was positively correlated with urinary N concentration (r = 0.96; p<0.001), and urinary GE:N (r = 0.54; p<0.001), but negatively correlated with BW (−0.32; p<0.001; Table 3). The urinary N concentration was positively correlated with urinary GE:N (r = 0.33; p<0.001) but negatively correlated with BW (r = −0.35; p<0.001). The urinary GE:N was negatively correlated with dietary CP (r = −0.30; p<0.001).

**Figure 1:**
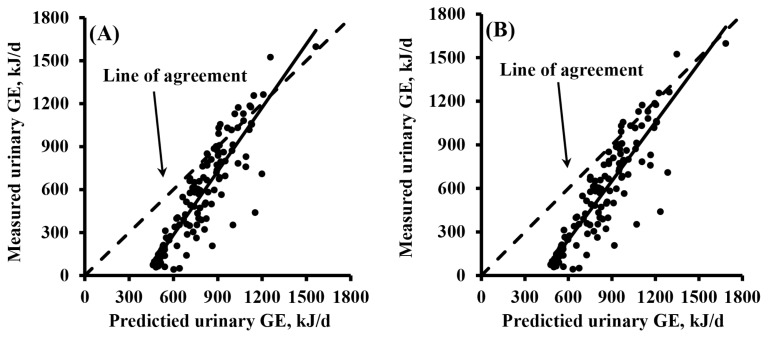
Validation of previous equations suggested by (A) Le Bellego et al [5] and (B) Le Goff and Noblet [6] for estimating urinary gross energy (GE) by urinary nitrogen. A total of 136 urine samples were used for the validation. Regression analyses were performed for measured minus predicted urinary GE adjusted to the mean as 0. (A) The slope for Le Bellego et al [5] was 0.48 (standard error = 0.06; p<0.001) and the intercept was −241.65 (standard error = 13.25; p<0.001). (B) The slope for Le Goff and Noblet [6] was 0.35 (standard error = 0.05; p<0.001) and the intercept was −285.00 (standard error = 13.25; p<0.001).

Based on the validation study, the slope for Le Bellego et al [5] (Eq. 1) was 0.48 (p<0.001) and the intercept was −241.65 (p<0.001; Figure 1) and the slope for Le Goff and Noblet [6] (Eq. 2) was 0.35 (p<0.001) and the intercept was −285.00 (p<0.001). These results indicate that both Eqs. 1 and 2 overestimated (mean bias; p<0.001) urinary GE concentrations and the overestimation was more pronounced for the low-GE urine samples (linear bias; p<0.001).

The novel equations developed for estimating urinary GE concentration in pigs were: urinary GE (kcal/kg) = −7.51+ 12.83×urinary N (g/kg) with r^2^ = 0.92 and p<0.001; −8.89+ 12.91×urinary N+0.022×BW (kg) with R^2^ = 0.93 and p<0.001; and −16.33+14.00×urinary N+0.192×BW−0.030×urinary N×BW with R^2^ = 0.92 and p<0.001 (Table 4).

## DISCUSSION

In pig diet formulations, ME and NE systems are widely employed [9], in which urinary energy output is considered as one of the unutilized energy excretions. Thus, an accurate determination of urinary GE concentration is essential for determining ME or NE of feeds fed to pigs. Although a direct determination of urinary GE concentrations using a bomb calorimeter has often been used, the drying procedure before the analysis is tedious and time-consuming [13]. Thus, researchers developed prediction models for estimating urine GE using N which can be determined without drying process [5,6,14]. The present study aimed to evaluate the previous prediction models and to develop novel equations.

In the validation study, linear and mean biases were observed in the previous equations [5,6], indicating that predicted values of urinary GE were overestimated particularly for low-GE urine. Although the reason for these results remains unclear, the biases would be due to several potential factors including pig BW, dietary CP concentrations, urine sampling procedures, and chemical analyses affecting urinary GE:N. The BW of pigs used for the development of previous equations ranged from approximately 60 to 70 kg [5,6] whereas nursery pigs weighing as light as 6 kg were included in the present study. The urine from younger pigs contains less creatinine and creatine expressed as total urinary N excretion compared with pigs of larger BW [15]. The relatively narrow and heavy BW range of the previous experiments [5,6] is likely one of the reasons for the overestimation of urinary GE. Creatinine and creatine, followed by urea, are the most abundant N-containing compounds in pig urine and have greater molecular weight-to-N ratios compared with urea. Consequently, the urine from nursery pigs would have less urinary GE:N compared with grow-finishing pigs.

In addition, the equations suggested in the present work may not be applicable to sows due to the potential influence of BW on urinary GE:N. The ranges of dietary CP and urinary N of the present study were wider than the previous experiments [5,6], which may also have affected the biases in the validation results. However, the procedures for the urine collection and chemical analysis were similar between the present and previous studies.

Among the chemical components in urine, only the urinary N concentration was used as an independent variable in the previous studies [5,6]. In ruminants, urinary carbon concentrations have also been used for estimating urinary GE based on the high correlation between carbon and GE concentrations [16,17], which is reasonable as organic compounds such as urea, creatinine, and creatine contain energy. With the same token, the N concentrations in urine are likely correlated with carbon. However, a model for estimating urinary GE using carbon may not be practically useful due to the difficulty in determining carbon concentrations compared with N analysis as suggested by Blaxter et al [16].

## CONCLUSION

The previous equations overestimate urinary GE particularly for low-GE urine of pigs. The novel prediction equations developed in the present study can fairly accurately estimate urinary GE concentration based on urinary N concentration and BW in pigs. Further research is warranted to validate and improve the novel equations in the future.

## Figures and Tables

**Table 1:** Prediction equations of urinary gross energy (GE) for growing pigs using urinary nitrogen (N) in the literature

Equation number^1)^	Prediction equation	r^2^	RSD
1	Urinary GE (kJ/d) = 28.4×urinary N (g/d)+425	0.95	-
2	Urinary GE (kJ/d) = 31.1×urinary N (g/d)+437	0.94	110

1) Eqs. 1 and 2 were reported by Le Bellego et al [5] and Le Goff and Noblet [6], respectively.

r^2^, coefficient of determination; RSD, residual standard deviation.

**Table 2:** Range and variability of urinary nitrogen (N) and gross energy (GE) concentrations in urine samples and body weight (BW) of pigs

Item^1)^	Mean	Minimum	Maximum	SD	CV (%)
Exp. 1 (n = 42)					
Urinary N (g/kg)	7.65	2.21	15.55	2.21	40.9
Urinary GE (kcal/kg)	93	15	187	43	46.4
Urinary output (kg/d)	0.44	0.20	1.21	0.22	50.4
Initial BW (kg)	7.0	6.0	8.7	0.8	11.8
Final BW (kg)	14.6	12.5	17.7	1.8	12.3
Exp. 2 (n = 94)					
Urinary N (g/kg)	4.19	1.06	12.48	2.12	51.2
Urinary GE (kcal/kg)	46	2	137	28	61.5
Urinary output (kg/d)	4.09	1.15	9.09	1.47	36.0
Initial BW (kg)	34.3	28.8	38.3	2.9	8.3
Final BW (kg)	99.6	89.3	111.4	5.8	5.8

1) The urine samples were obtained from barrows (Landrace×Yorkshire) fed 18 diets in 2 experiments [7,8]. The concentrations of crude protein in the experimental diets ranged from 8.0% to 22.7% (as-is basis).

SD, standard deviation; CV, coefficient of variation.

**Table 3:** Correlation coefficients among urinary nitrogen (N, g/kg), urinary gross energy (GE, kcal/kg), urinary GE-to-N ratio (GE:N), body weight (BW, kg), and dietary crude protein (g/kg) in 136 urine samples

Item	Urinary GE	Urinary N	Urinary GE:N	BW
Urinary N	0.96^***^			
Urinary GE:N	0.54^***^	0.33^***^		
BW	−0.32^***^	−0.35^***^	−0.01	
Dietary crude protein	−0.01	0.09	−0.30^***^	0.12

*** p<0.001.

**Table 4:** Novel equations for estimating urinary gross energy (GE, kcal/kg) of pigs (n = 136)

Item	Intercept	Independent variables			RMSE	R^2^	p-value

		Urinary N (g/kg)	BW (kg)	Urinary N×BW			
Eq. 3	−7.51^***^ (1.98)	12.83^***^ (0.33)	-	-	11.21	0.918	<0.001
Eq. 4	−8.89^**^ (3.00)	12.91^***^ (0.36)	0.022 (0.034)	-	11.24	0.928	<0.001
Eq. 5	−16.33^***^ (4.16)	14.00^***^ (0.56)	0.192^*^ (0.076)	−0.030^*^ (0.012)	11.02	0.922	<0.001

Values in parentheses are standard errors.

* p<0.05,

** p<0.01,

*** p<0.001.

N, nitrogen; BW, body weight; RMSE, root mean square error; R^2^, coefficient of determination.
